# Review of Phosphorus-Based Polymers for Mineral Scale and Corrosion Control in Oilfield

**DOI:** 10.3390/polym14132673

**Published:** 2022-06-30

**Authors:** Yuan Liu, Ping Zhang

**Affiliations:** Department of Civil and Environmental Engineering, Faculty of Science and Technology, University of Macau, Taipa 999078, Macau; yb87401@connect.um.edu.mo

**Keywords:** phosphorus, polymers, mineral scale, corrosion, oilfield

## Abstract

Production chemistry is an important field in the petroleum industry to study the physicochemical changes in the production system and associated impact on production fluid flow from reservoir to topsides facilities. Mineral scale deposition and metal corrosion are among the top three water-related production chemistry threats in the petroleum industry, particularly for offshore deepwater and shale operations. Mineral scale deposition is mainly driven by local supersaturation due to operational condition change and/or mixing of incompatible waters. Corrosion, in contrast, is an electrochemical oxidation–reduction process with local cathodic and anodic reactions taking place on metal surfaces. Both mineral scaling and metal corrosion can lead to severe operational risk and financial loss. The most common engineering solution for oilfield scale and corrosion control is to deploy chemical inhibitors, including scale inhibitors and corrosion inhibitors. In the past few decades, various chemical inhibitors have been prepared and applied for scaling and corrosion control. Phosphorus-based polymers are an important class of chemical inhibitors commonly adopted in oilfield operations. Due to the versatile molecular structures of these chemicals, phosphorus-based polymeric inhibitors have the advantage of a higher calcium tolerance, a higher thermal stability, and a wider pH tolerance range compared with other types of inhibitors. However, there are limited review articles to cover these polymeric chemicals for oilfield scale and corrosion control. To address this gap, this review article systematically reviews the synthesis, laboratory testing, and field applications of various phosphorus-based polymeric inhibitors in the oil and gas industry. Future research directions in terms of optimizing inhibitor design are also discussed. The objective is to keep the readers abreast of the latest development in the synthesis and application of these materials and to bridge chemistry knowledge with oilfield scale and corrosion control practice.

## 1. Introduction

Enhanced oil recovery (EOR) technology such as water/polymer/CO_2_ flooding has been widely adopted over several decades in the petroleum industry to enhance the hydrocarbon recovery rate [1,2]. The two major water-related operational challenges associated with EOR are mineral scale deposition and metal corrosion [3]. The onset of these challenges is due to the changes in operational conditions as a result of a set of complicated chemical reactions from reservoir to processing facilities [4,5]. Deposited mineral scales (i.e., CaCO_3_, BaSO_4_, Ca_3_(PO_4_)_2_, etc.) and corrosion products (i.e., CuO, Fe_2_O_3_, Fe_3_O_4_, ZnO, etc.) can cause a reduction in formation porosity, a decline in productivity, wellbore damage, and a significant financial loss [6,7,8]. Furthermore, mineral scaling and corrosion can impair the integrity of the operational systems, leading to severe threats to personnel safety. Scales are insoluble crystalline inorganic precipitates from the water phase where the water is oversaturated with the scaling ions due to the change in operating conditions and/or mixing of incompatible waters [9,10,11]. Scale solubility is the primary driving force for scale formation, which can affect the kinetics and thermodynamics of the scale formation [12,13]. The major factors influencing scale solubility include the composition and concentrations of scaling ions, temperature, ionic strength, etc. [14,15]. As for the corrosion process, material surfaces are gradually destroyed by interactions with an aggressive medium (such as saline water), which will form corrosion products, including sulfides and other scales such as carbonates and hydroxides [16]. The most commonly encountered gases associated with corrosion include H_2_S, CO_2_, and O_2_ [17].

To overcome these issues, the most cost-effective and widely adopted engineering solution for controlling scale deposition and corrosion is the application of chemical inhibitors, including scale inhibitors and corrosion inhibitors [18,19,20]. Scale inhibitors are chemical substances, such as the phosphonates, polyacrylates, and poly-maleates, added in a low dose (typically in the level of a few mg L^−1^) to the brine to delay or retard the formation of scale [21,22,23]. Some polymeric scale inhibitors, such as polyaspartate and polyepoxysuccinic salts, can also have corrosion inhibition properties in addition to their more common use as a scale inhibitor [24]. Conventional scale inhibitors commonly adopted in the oil field are summarized in Table 1.

Among these inhibitors, inorganic phosphates and phosphonates are known to have strong complexing ability with a wide variety of metal ions due to their high versatility in their protonation states, such as monoprotonated R-PO_3_H^−^ and fully deprotonated R-PO_3_^2−^ [25,26]. Most of the phosphonate inhibitors have shown a better performance than the polymeric scale inhibitors [10]. At the same time, phosphorus is easy to be measured by optical emission microscopy or the colorimetric method with high accuracy [11,27,28]. These traditional scale inhibitors, particularly those based on phosphate and phosphonates with a high phosphorus content, are reported to be excellent inhibitors with a satisfactory inhibition efficacy [29,30]. Phosphonates and their derivatives were originally used as scale inhibitors. Interestingly, some of these chemicals were later used as very effective and successful corrosion inhibitors in many practical applications [31,32,33]. However, these inhibitors can also form an undesirable phosphate scale with high calcium concentrations. In addition, due to environmental concerns, there has been a growing interest in applying environmentally friendly inhibitors such as polymeric inhibitors [34,35,36]. Nevertheless, it is known that some “green” inhibitors (e.g., polymaleic acid and polyaspartic acid) are not always as effective in impeding scale formation. Moreover, it should be noted that polymers, especially those composed of only C, H, and O elements, are difficult to measure accurately in an oil field, especially at a low level of concentration of a few mg L^−1^ [37,38]. Thus, it is necessary to seek an acceptable balance between inhibition ability and environmentally friendly properties. The phosphonate-tagged polymers provide an opportunity to diminish phosphorus abundance in scale inhibitor molecules. At the same time, it is worth noting that the introduction of a phosphonic acid moiety into a polycarboxylate chain can result in a higher chemical manufacturing cost than adopting polycarboxylate/phosphonate blends for scale control, but it provides more benefits compared with the blended chemicals [39,40]. In the past few decades, incorporation of phosphorus-containing functional groups into a polymer backbone has been an ever-increasing approach to improving inhibition efficiency in oilfield scale and corrosion control [41,42,43]. These products combine the attributes of both polymers and phosphonates with the advantages of a higher thermal stability, higher calcium tolerance, and wider pH tolerance range compared with other types of inhibitors [44,45,46]. Kaseem et al. [47] reported that phosphoric acid functionalized polyvinyl alcohol provided superior corrosion protection properties for Mg alloy substrates.

**Scheme 1:**
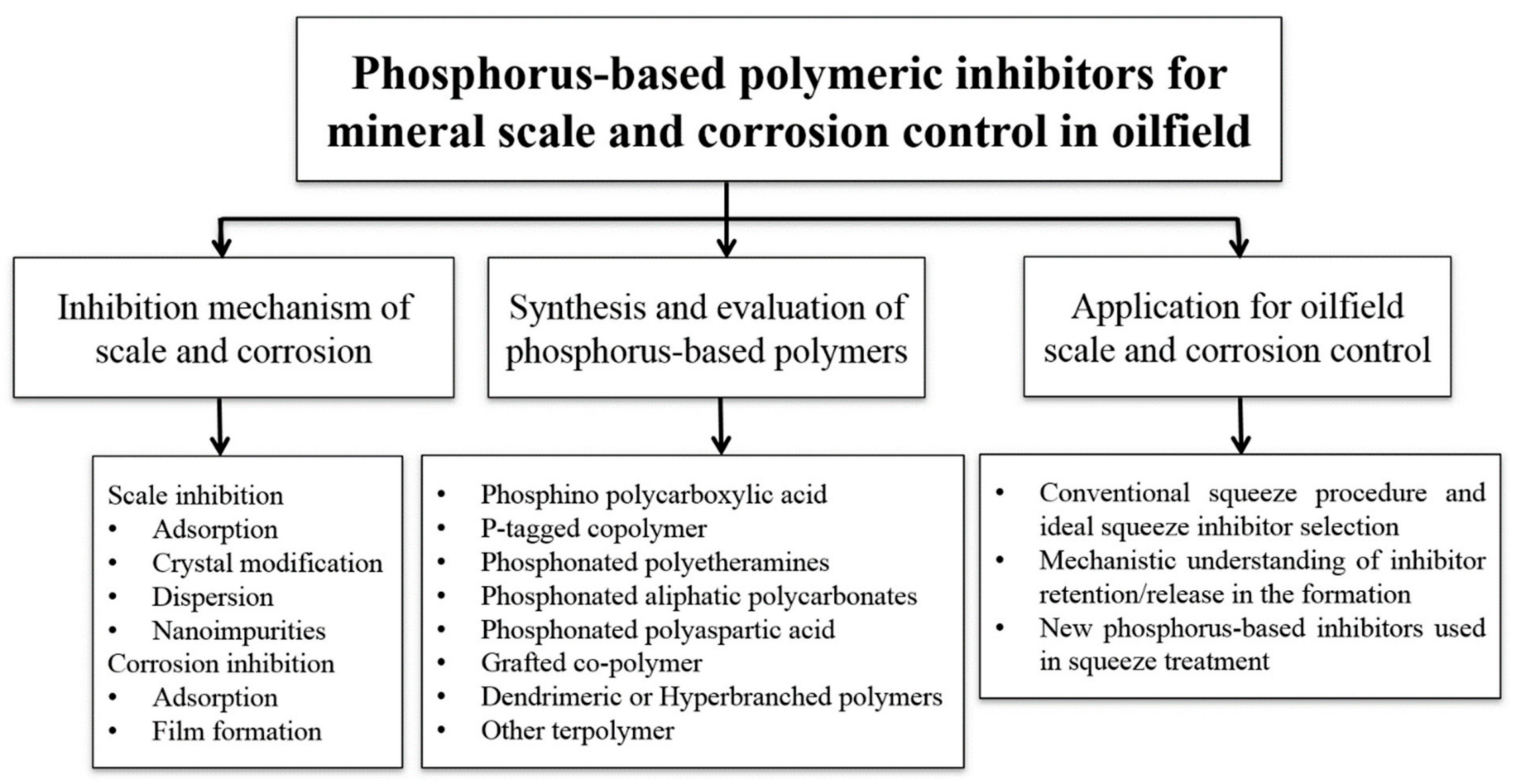
Structure of this review article.

Although a number of existing review articles provide a wide overview on the topic of oilfield chemical inhibitors [18], such as organophosphonic acids [21], green inhibitors [34], and inhibitor nanomaterials [22], as well as polymers as corrosion inhibitors [48], there is no review articles focusing on phosphorus-based polymers for applications of mineral scale and corrosion control in an oilfield. This review presents relevant work achieved to date on phosphorus-based polymers for potential mineral scale and corrosion control applications. To the best of our knowledge, this is the first report to focus on the review of the phosphorus-based polymers for mineral scale and corrosion control in oilfield operations. The structure of this review article is presented in Scheme 1.

## 2. Chemical Inhibition Mechanism

### 2.1. Scale Inhibition

There are a number of principal mechanisms responsible for the inhibitory effectiveness of scale inhibitors in preventing or retarding scale formation [49]. These mechanisms include nucleation inhibition, crystal growth inhibition, scale dispersion, and crystal modification [50,51,52]. Hoang [53,54] has provided a comprehensive review of mechanisms of scale formation and inhibition. Oshchepkov and Popov [55] pointed out the drawbacks of these conventional inhibition mechanisms and proposed an alternative view that a scale inhibitor inhibits the scale formation by blocking the surface of “nanodust” particles rather than the generally assumed interference with the formation of homogeneous clusters. The mechanisms of how inhibitors inhibit scale formation have been studied for many years. However, a comprehensive inhibition mechanism has not yet been established, and the details of fundamental mechanisms accounting for inhibitory effect are yet to be clarified. In-depth understanding of the inhibition mechanism not only benefits the inhibition efficiency and their performance for scale control in oilfield operations but also facilitates the conceptual design of novel inhibitors embedded with various functional moieties.

#### 2.1.1. Adsorption 

It is widely accepted that the key mechanism accounting for inhibitor effectiveness is that the inhibitors can adsorb onto the growth sites of the nuclei and block active growth sites [26,56,57]. Inhibitor adsorption is considered a fundamental role in the scale prevention and control process [58,59,60]. Some corrosion inhibitors adsorb onto metal surfaces to form a protective layer, preventing the interaction between the corrosive fluids and the metal via a barrier coating [61]. At low inhibitor concentrations, the inhibitor would be adsorbed on the mineral surface, which can be characterized by adsorption isotherms such as a Langmuir-type isotherm and a Freundlich-type isotherm [11,26]. Tomson et al. [50] proposed that hydrophobic repulsion of neutral inhibitors for macromolecules from liquid solutions is responsible for inhibitor adsorption. In the presence of divalent metals such as barium, calcium, iron, and magnesium, inhibitor effectiveness can be significantly enhanced because of the formation of a metal–inhibitor complex, especially for Ca–inhibitor [62,63]. Xiao et al. [64] found out that only metal-complexed phosphino-polycarboxylic acid (PPCA) can effectively inhibit barium sulfate (barite) scale formation, and un-complexed PPCA cannot. Therefore, it is necessary to understand the acid–base and complexation equilibria of inhibitors and various divalent metal ions. Previously, acid–base chemistry of phosphonates and their complexation equilibria have been described by an electrostatic-type equation [65,66,67]. Because organic inhibitors are usually weak polymeric acids, factors such as temperature, pH, ionic strength, and ionic composition of brine solution can affect their speciation in solution. Similar to phosphonates, a polymer-based inhibitor can be simplified as a hypothetical monoacid with the same concentration of its monomer. For example, Xiao et al. [68] systematically studied solution speciation of PPCA in an aqueous solution as a function of the aforementioned physiochemical parameters and Ca concentration with a combination of electrostatic theory and potentiometric titrations. This metal–inhibitor complex can easily adsorb onto the mineral scale surface, resulting in a profound impact on inhibiting nucleation/crystal growth as well as the efficiency of inhibitors [63,65].

#### 2.1.2. Crystal Modification 

**Figure 1:**
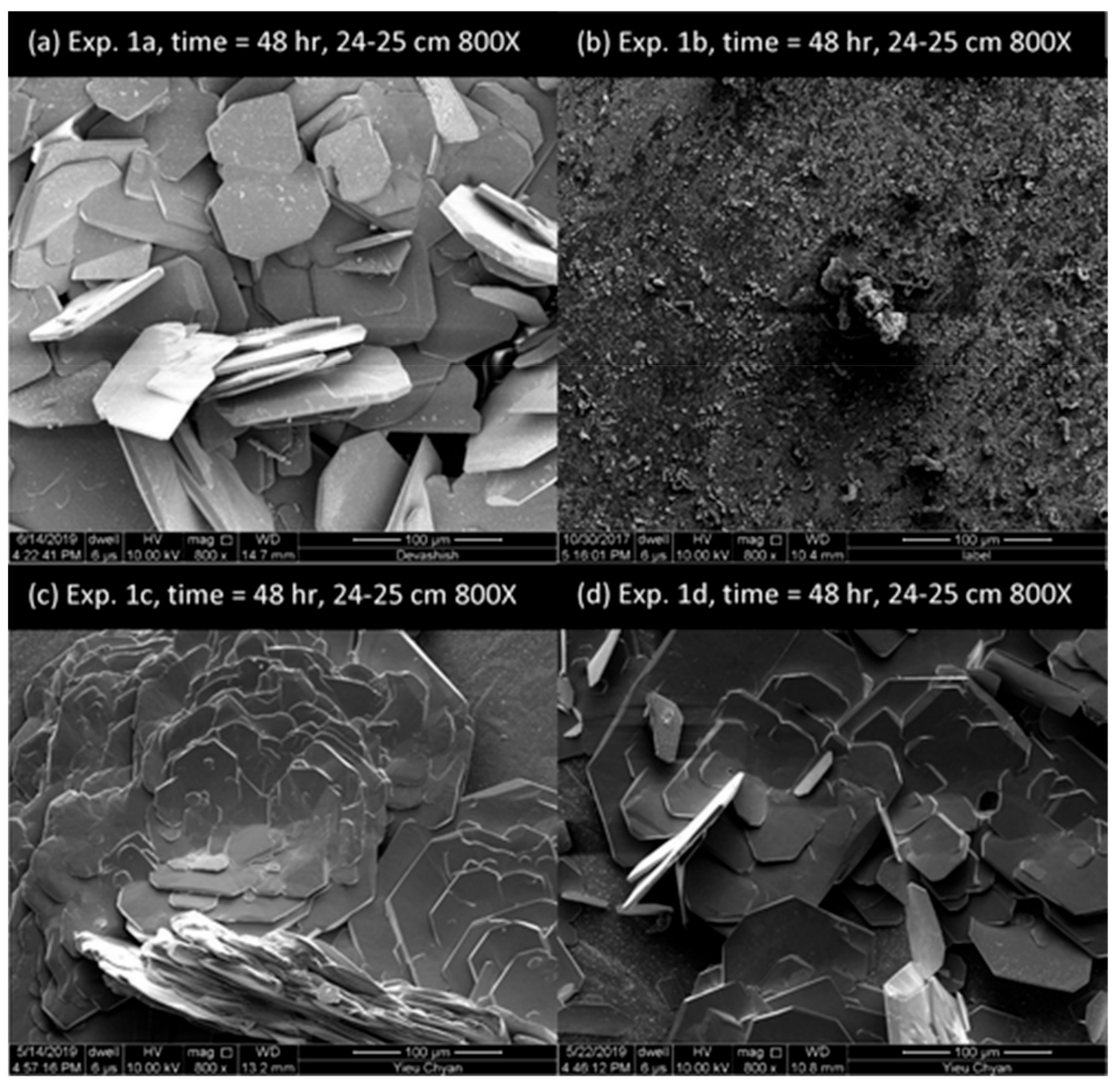
SEM image of stainless-steel tubing in experiments: (**a**) without the presence of inhibitors, (**b**) tubing wall with no deposited barite crystal, (**c**) presence of PPCA, and (**d**) SPCA [70].

A fraction of inhibitors can inhibit scale formation by crystal modification. Complexing with foreign ions, inhibitors can adsorb onto the growth sites and insert into the scale lattice that modifies the structure and diminishes the adhesion of scales to solid surfaces [7,51]. For example, the inhibitor complexed with Ca^2+^ inhibits barite formation and has the ability to incorporate into the barite lattice and change its morphology [51]. Moreover, the results disclose that up to approximately 6% Ba^2+^ in barite can be substituted by Ca^2+^. Pina et al. [69] investigated the initial stages of barite formation in the presence of phosphonate inhibitors. The morphology changes of the deposited barite were directly observed using an atomic force microscope. They found that the presence of the inhibitor in solution not only reduced the barite growth rates but also influenced its morphology in some cases. The typical shape of islands on a barite (001) plane was clearly changed; the islands became irregular with rounded corners. In another study, Lu et al. [70] evaluated scale inhibitors of various types, including diethylenetriamine-pentamethylene phosphonic acid (DTPMP), PPCA, and sulfonated poly(carboxylic acid) (SPCA) for barite deposition inhibition in a dynamic flow loop apparatus. The morphology of deposited barite crystal impacted by the presence of inhibitors was also observed using scanning electron microscopy (SEM). According to the SEM images, the morphology of barite changes from sharp-edge rosette (Figure 1a) to round-edge rosette (Figure 1c) with the presence of PPCA, whereas the presence of SPCA (Figure 1d) did not noticeably change the crystals’ morphology. However, there is a lack of direct correlation between inhibition efficacy and crystal distortion. Some other authors have revealed that the least efficient scale inhibitors, instead of the most efficient inhibitors, have a higher tendency to change the morphology of scale crystals [71,72]. More investigations should be conducted to clarify the crystal modification mechanism. 

#### 2.1.3. Dispersion

Some scale inhibitors also have a dispersion effect on the formed scales, especially for organic polymeric inhibitors [73]. Inhibition by dispersion is an indirect inhibition mechanism that prevents formed small-scale particles from gathering and precipitating by repulsive electrostatic charges [74]. This mechanism involves the chemical reduction of agglomeration and settling of suspended particles, which is often used in sulfide scale control [14,75]. For instance, Amjad [76] investigated several polymeric and non-polymeric scale inhibitors as iron oxide dispersants in an aqueous system. They found that increasing dispersion time and additive concentrations can increase the dispersion level of iron oxide by phosphonates and polymeric additives. It is worth noting that among the five phosphonates as iron oxide dispersants tested by these authors, polyamino polyether methylene phosphonic acid (PAPEMP) exhibited excellent performance on dispersing iron oxide with the addition of neutral moiety, i.e., polyether, showing the improvement of the dispersing ability of PAPEMP.

#### 2.1.4. Nanoimpurities’ Role in Scale Formation/Inhibition

More recently, a non-conventional inhibition mechanism in which scales are inhibited by heterogeneous formation on a “nanodust” particle surface, instead of ion pairs or clusters formed via homogeneous nucleation, was investigated [55]. Several fluorescent-tagged scale inhibitors were synthesized, which were able to provide visualization of the functionality of scale inhibitors during the formation of gypsum and some other scales [77,78,79]. From these studies, it appeared that, in many cases, scale inhibitors are not all located on the surface of sparingly soluble salt crystals, but rather form their own solid phases. Therefore, formation of scale particles could still be inhibited by the scale inhibitor, although a fraction of the inhibitors are not directly interacting with crystal surface. This paradox can be explained by the role of natural nanoimpurities in scale formation/inhibition. In the supersaturated solutions of sparingly soluble salts, nanoimpurities can act as crystallization centers by serving as the templates or seeds for crystallization. The scale inhibitors prevent the scaling ions from attaching to the surface of these nanoimpurities and hereby delay the crystallization process [80].

### 2.2. Corrosion Inhibition

From a physical–chemical point of view, the role of corrosion inhibitors can be divided into two main types: adsorption type and film-forming type [81,82]. Similar to the adsorption mechanism for mineral scale inhibitors, corrosion inhibitors can adsorb onto the metal surface as a passivating film (e.g., phosphates and polyphosphates) through physical or chemical adsorption. The commonly reported adsorption mechanism is electrostatic attraction or forming a coordinate covalent bond between the corrosion inhibitor and the metal surface [83,84]. This nonreactive thin surface film serves as a barrier to restrict access of corrosive substances and protect the metal against further corrosion [61,85]. Generally speaking, formation of a passivating film is a special case of adsorption. Phosphates such as Na_2_HPO_4_ or Na_3_PO_4_ can react with Fe^3+^ in the presence of dissolved oxygen to form an insoluble protective passive Fe_3_O_4_ film, which inhibits iron corrosion [86]. However, it should be noted that all passivation inhibitors will accelerate corrosion if the dose is insufficient [87]. Film-forming corrosion inhibitors are more preferred to be applied in downhole or wellhead [88]. Most of them are organic amphiphiles containing both polar (i.e., a hydrophilic headgroup) and nonpolar (i.e., a hydrophobic tail) regions [89]. They can form an oily layer or film on the surface, which acts as a hydrophobic barrier against the acid gases (e.g., CO_2_ and H_2_S) [90].

The mechanisms of scale inhibition by chemical inhibitors include conventional mechanisms, such as nucleation inhibition, crystal growth inhibition, scale dispersion, and crystal modification, as well as unconventional mechanisms of nanoimpurities. Corrosion inhibitors can function via passivating (anodic) and film forming mechanisms.

## 3. Synthesis and Evaluation of Phosphorus-Based Polymers

### 3.1. Introduction

**Figure 2:**
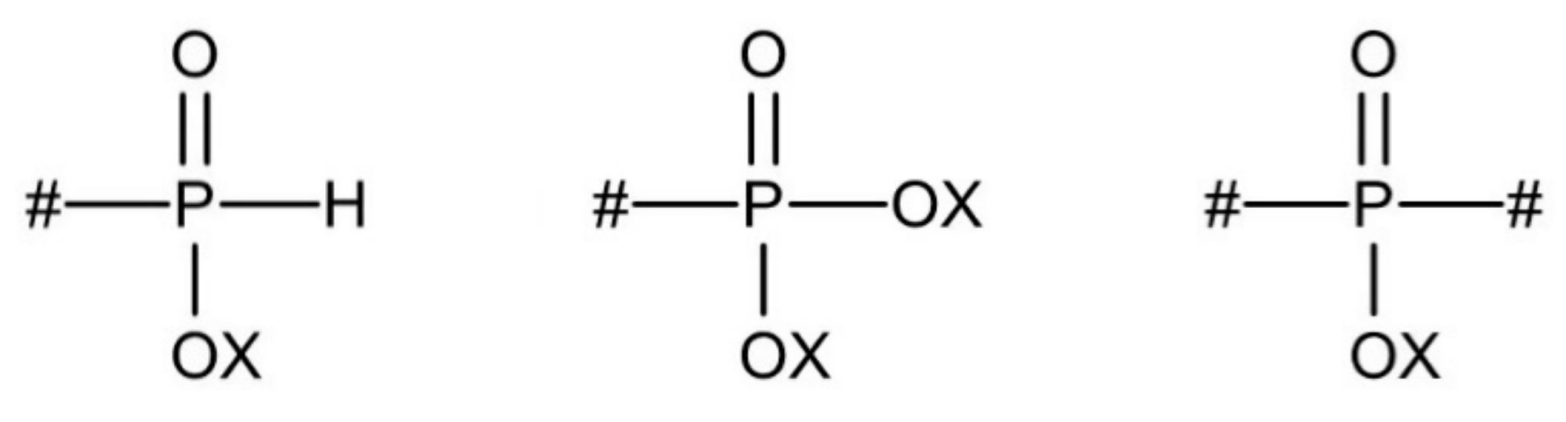
Common structures of phosphorus-containing units in polymers.

During the last three decades, many phosphorus-based polymers with different compositions and structures have been developed and investigated as mineral scale and corrosion inhibitors for oilfield applications. Mady [91] provided an overview of various approaches to synthesis oilfield scale inhibitors. Some common phosphorus-based polymeric inhibitors are summarized in Table 2. The common structures of phosphorus-containing units in the polymer composition are shown in Figure 2, wherein # represents a binding site to a polymer residue, and X denotes one of the species of hydrogen, monovalent cations, or monovalent equivalents of polyvalent cations.

**Figure 3:**
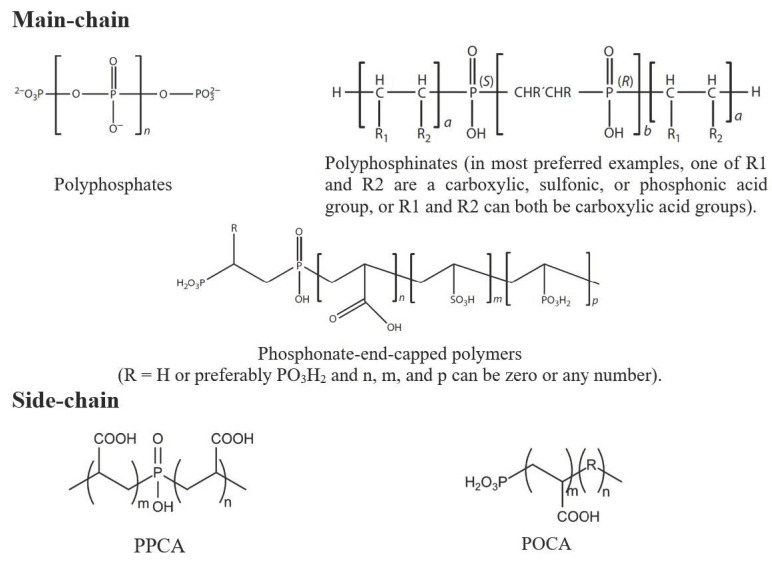
Structures of typical phosphorus-containing polymers.

According to the position of the phosphorus-containing group in the polymer, it can be divided into two types of main-chain and side-chain, with selected examples shown in Figure 3.

More recently, different varieties of new-style phosphorus-containing polymer inhibitors have been synthesized, such as phosphorus end-capped polymers, grafted copolymers, hyperbranched polymers, etc. These attempts to make more environmentally friendly and biodegradable inhibitors containing phosphonic acid groups for oilfield application have been increasingly reported. An introduction of emerging phosphorus-based polymers and their corresponding laboratory (or industrial) synthesis approaches is presented below. 

### 3.2. Phosphino-Polycarboxylic Acid

PPCA is one of the most commonly adopted phosphino polymers in the oil industry, and it comprises a single phosphino group bridging two polyacrylic or polymaleic chains [92]. There are several patents published on synthesis procedures of PPCA inhibitors [93]. Typically, PPCA is synthesized by free radical polymerization of unsaturated organic carboxylates such as acrylic acid and maleic acid with hypophosphorous compounds/acid [94]. Recently, Malaie et al. [95] reported the synthesis of PPCA copolymer by a simplified method according to free radical polymerization of phosphinic acid monomers and acrylic acid, and these authors adopted the prepared PPCA inhibitor for gypsum scale inhibition. 

### 3.3. Phosphorus-Tagged (P-Tagged) Copolymer

**Figure 4:**
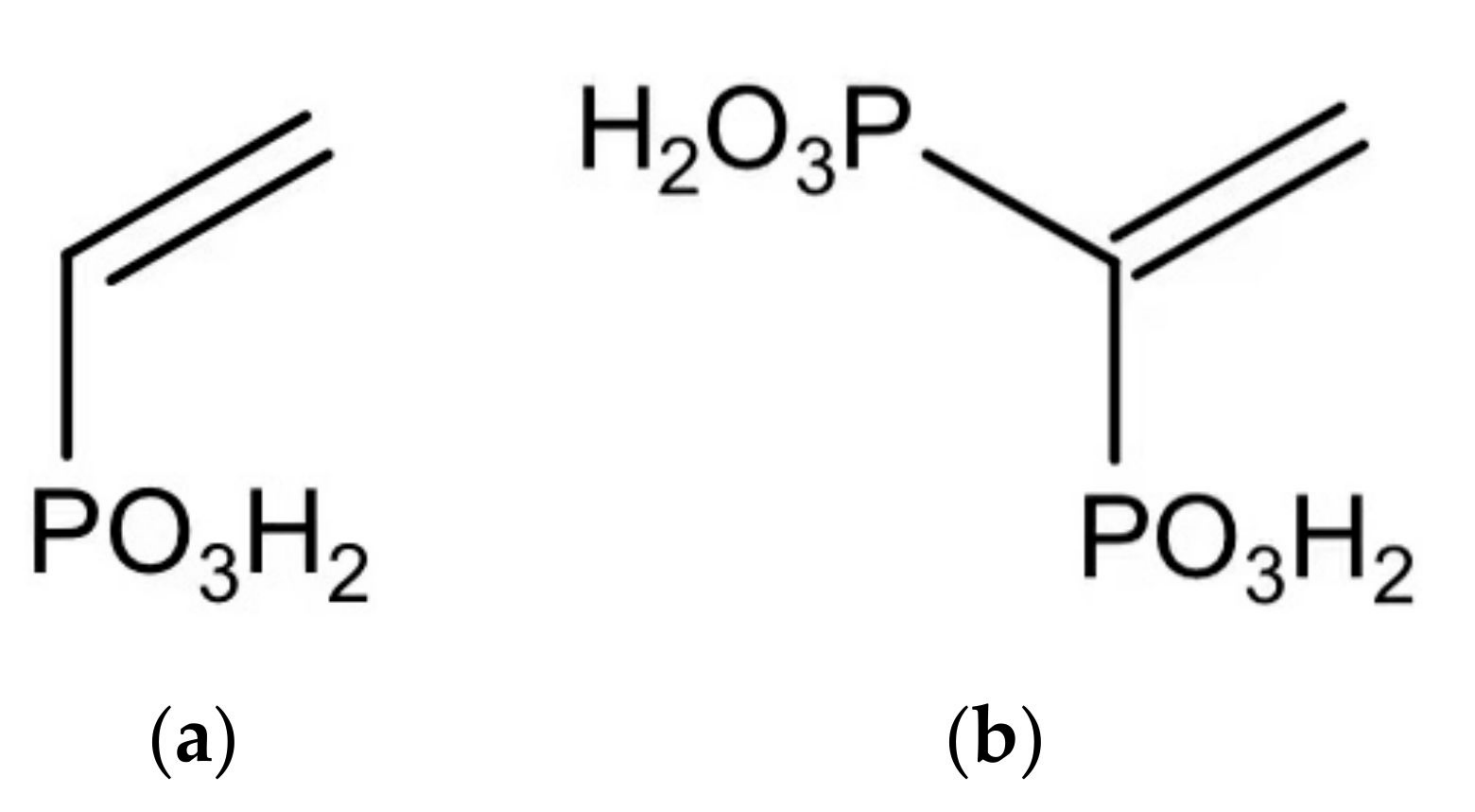
Phosphorus based vinylic monomer species: (**a**) VPA and (**b**) VDPA.

There have been many efforts to place phosphorus-containing species in the tail position of the backbone of an existing polymer [96,97,98,99]. Many phosphorus-based vinylic monomer species, such as vinyl phosphonic acid (VPA) and vinylidene diphosphonic acid (VDPA) (Figure 4) [100,101], are only used as end-capping monomers to make polyvinyl polymers due to their higher costs compared to other common monomers, such as carboxylic acid (i.e., methacrylic acid, maleic acid) and sulfonic acid (i.e., vinylsulfonic acid). For instance, Davis et al. [102,103] reported the synthesis of a range of polymeric scale inhibitors using VDPA as a starting point for chain-growth polymerization. Because of the additional phosphonate groups, these end-cap polymers demonstrate a number of advantages for scale control, including enhanced adsorption properties and increased stability under extreme high temperature conditions (e.g., up to 200 °C). Table 3 summarizes the P-tagged copolymers reported previously. Campo et al. [104] also listed a number of structures of end-capping phosphorus polymers.

For other methods of preparing tagged polymers, Wang et al. [97] prepared a low phosphonic copolymer involving reacting maleic anhydride (MA) with sodium p-styrene sulfonate (SS) in a redox environment of hypophosphorous and hydrogen peroxide as initiators. These authors claimed that carboxylic or phosphonate anions of this low phosphonic copolymer could bind to calcium ions at calcium carbonate (calcite) surfaces. Based on laboratory investigation, it was noticed that the scaling inhibition performance of this fabricated copolymer was excellent with a reduced inhibitor dosage. The improved inhibition effects are attributed to the adsorption of the low phosphonic copolymer on the crystal surface causing a deformation of the crystal morphology.

### 3.4. Phosphonated Polyetheramines 

**Figure 5:**
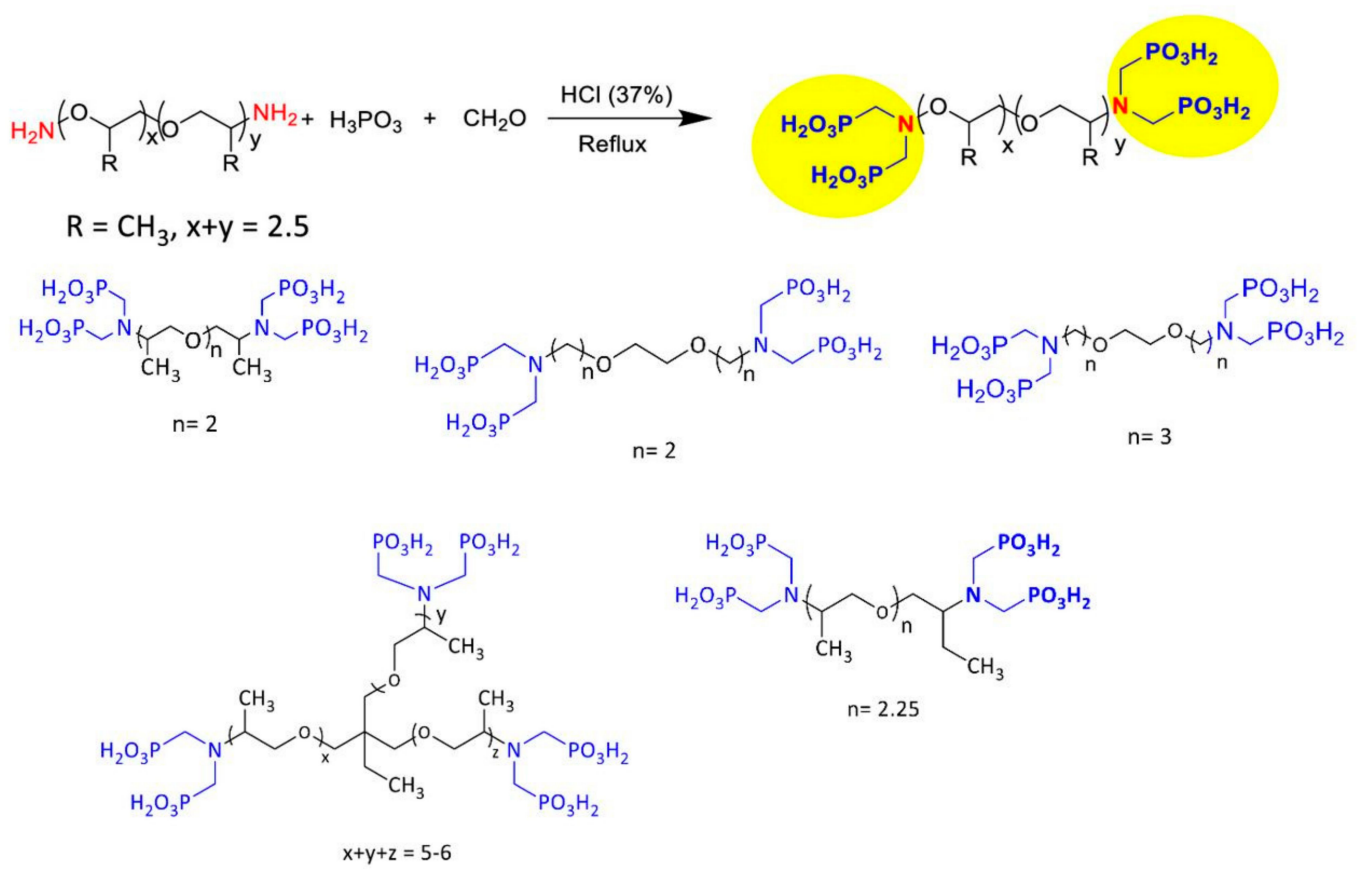
Synthesis and chemical structure of phosphonated polyetheramine derivatives [108].

Polyethers are polymers that are formed by the combination of monomers through ether (C−O−C) linkages. Because of their unique physical, chemical, and biodegradation properties, it is a very important group of inhibitors for biological and industrial applications. It has been found that PAPEMPs have a strong ability to tolerate high calcium levels and possess excellent inhibition property against calcite scale, even under harsh conditions, such as an elevated solution pH, a high calcite saturation state, and a high dissolved solids concentration. For example, Chen and Matz [105] reported a series of PAPEMPs as scale inhibitors for calcite control at a high pH level with the structure of inhibitor molecule shown in Table 2. According to these authors, the PAPEMP inhibitors demonstrated a satisfactory inhibition of CaCO_3_ scale under pH 8.5−9.5. This class of polyether scale inhibitors have been produced commercially by several chemical vendors [105,106,107]. Very recently, Mady et al. [108] reported the development of a series of linear and branched phosphonated polyetheramines and applied them for downhole oilfield calcite or barite scale control at low solution pH conditions. These phosphonated polyetheramines with linear and branched structures were prepared by the Moedritzer−Irani reaction, which involves the addition of polyether amines introduced into the reaction with formaldehyde and phosphorous acid in strongly acidic conditions. Figure 5 illustrates the general synthesis route of this phosphonated polyetheramine scale inhibitor and the chemical structures of five common phosphonated polyetheramines.

According to these authors, phosphonated modified polyetheramines not only have an improved inhibition performance against both calcite and barite scales compared to common commercial phosphonated scale inhibitors, but also show superior calcium tolerance, thermal stability, and seawater biodegradability.

### 3.5. Phosphonated Aliphatic Polycarbonates 

**Figure 6:**
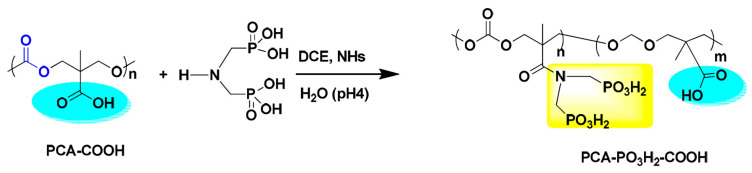
Functionalization of PCA-COOH with phosphonate groups to PCA-PO_3_H_2_-COOH [109].

Recently, Mady et al. [109] reported the synthesis of modified aliphatic polycarbonates with phosphonate groups for carbonate and sulfate scale control in an oilfield as an attempt to seek a new class of environmentally friendly scale inhibitors. This approach involves a 5-step reaction route. The prepared trimethylene carbonate-COOH reacts with 2-iminodimethyl-phosphonic acid at pH 4.7. After it was dialyzed and concentrated under vacuum, precipitation occurred in the residual solution with ca. pH 4. The precipitate of the biphosphonated product with a molecular weight of 3800 g mol^−1^ was separated by centrifugation and dried under vacuum. Figure 6 shows the synthesis of aliphatic polycarbonates blended phosphonate groups (PCA-PO_3_H_2_-COOH). The results showed that these modified aliphatic polycarbonate coupled phosphonate copolymers displayed enhanced thermal stability and biodegradation performance. 

### 3.6. Phosphonated Polyaspartic Acid

**Figure 7:**
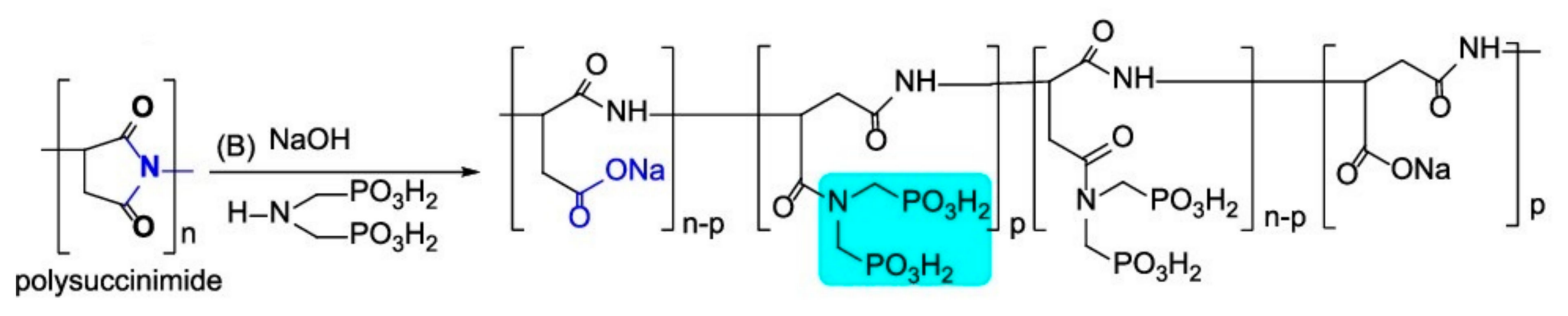
Synthesis of modified polyaspartic acid with aminomethylene phosphonic acid [44].

Polyaspartic acid (PASP) belongs to a class of polyamino acids. Because the peptide bonds on its structural backbone are easily broken up, PASP is thus biodegradable [110]. The final degradation products of PASP include ammonia, carbon dioxide, and water, which are harmless to the environment. Therefore, PASP is widely used in water treatment, medicine, agriculture, and other industries [111]. As a scale inhibitor, it not only has a good inhibition performance against the formation of carbonates and sulfates scale, but also possesses a dispersing effect, and can effectively prevent corrosion of metal surfaces [112,113]. However, hydrolysis of PASP occurs easily at high temperatures (higher than 85 °C), which will reduce the activity of PASP and lead to lower efficacy inside the reservoir. Mady et al. [44] developed a new class of coupled phosphonate with PASP to inhibit the CaCO_3_ and BaSO_4_ scales for squeeze treatment applications under high pressure and high temperature conditions. A series of modified PASPs with phosphonate or sulfonate were synthesized via aminolysis of polysuccinimide with nucleophilic amine reagents under alkaline conditions. Figure 7 shows the synthesis reaction of modified PASP with aminomethylene phosphonic acid. These coupled phosphonic acid and bisphosphonic acid products were also investigated for their chemical tolerance against high calcium levels and long-term thermal aging. Researchers found that PASP-capped aminomethylene phosphonic acid provided an excellent calcite scale inhibition property and displayed a desirable thermal stability under harsh oilfield conditions (at 130 °C for 7 days) in comparison to PASP and other modified compounds such as PASP-capped bisphosphonic acid and PASP-capped aminoethanesulfonic acid. This phosphonated polymer offered a remarkable calcium tolerance ability with Ca^2+^ ions up to 100 mg L^−1^, and exhibited a moderate inhibition effect within the tested Ca^2+^ ions range of 1000 to 10,000 mg L^−1^.

### 3.7. Grafted Copolymer

**Figure 8:**
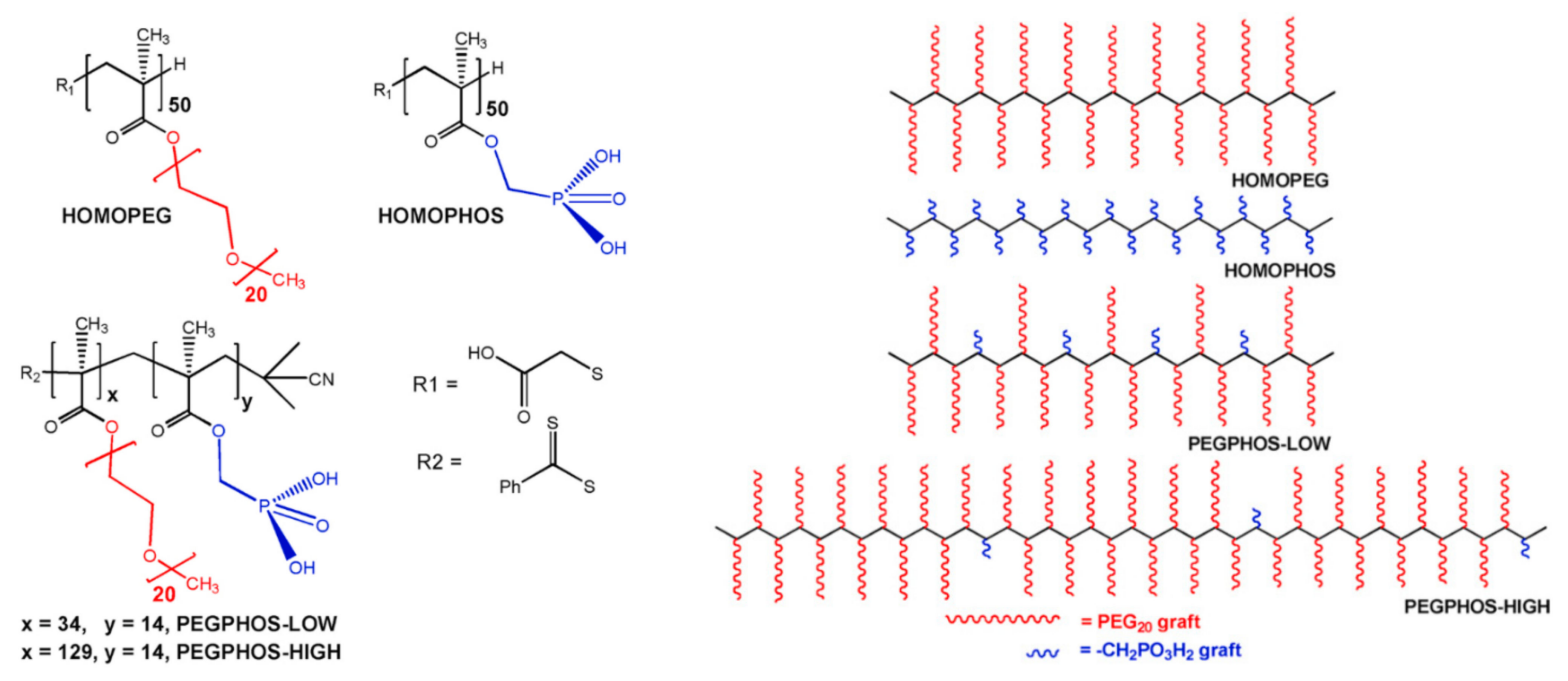
Schematic structures of methacrylate-based polymeric scale inhibitors with phosphonate and PEG grafts [117].

Graft copolymer is a type of copolymer that consists of a main chain and one or more branched chains linked to it by covalent bonds. With biodegradability and other advanced properties, they have been widely used in the petroleum industry as corrosion inhibitors, flocculants, and thickening agents [114,115,116]. Spinthaki et al. [117,118] have prepared interrelated methacrylate-structured polymers, including the grafting of phosphonic acid (PHOS) to a methacrylate acid side-chain or with grafting of polyethylene glycol (PEG), as shown in Figure 8. These authors evaluated these polymeric scale inhibitors with eight different types of scales, including amorphous silica, carbonates, silicates, and sulfides. It was reported that the most efficient inhibitor is PEGPHOS-LOW (containing 14 phosphonate grafts and 34 PEG grafts) with a dual scale control function including enhanced solubility of scaling species and improved dispersibility of scale particles. These authors subsequently tested these inhibitors in artificial geothermal brines of variable stress, containing all above scales together. However, they showed a poor inhibition performance with brines in high scaling tendency systems (with the addition of 500 mg L^−1^ Ca^2+^ and 750 mg L^−1^ CO_3_^2−^).

### 3.8. Dendrimeric or Hyperbranched Polymers

**Figure 9:**
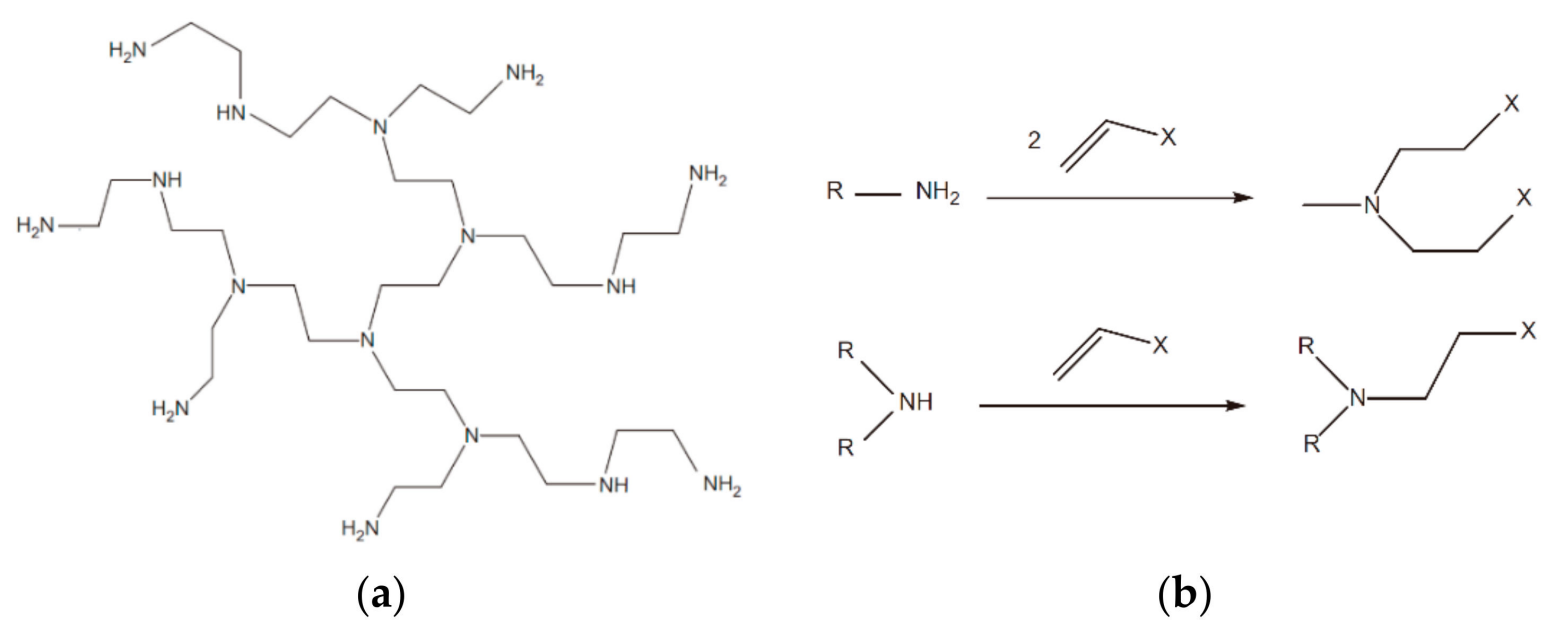
(**a**) Generalized structure of a hyperbranched polyethyleneimine, showing primary, secondary, and tertiary amine groups; (**b**) addition of vinyl monomers to primary and secondary amines. X = phosphonate, sulfonate, or carboxylate [122].

Polymers can be generally classified into three groups according to their structures: linear, branched, or crosslinked [119]. Hyperbranched polymers are a special type of dendritic polymers with high levels of branched spherical structures, which have a wide range of application prospects and have attracted a growing interest in polymer science [43,120,121]. Because of its high aqueous solubility, low viscosity, and branched structure with easily modified terminal groups, there is a possibility to choose hyperbranched polymer as a new type of scale and corrosion inhibitor, as this chemical can be easily adsorbed onto mineral solid surfaces or metal layers in aqueous solution. Jensen and Kelland [122] investigated the synthesis of a series of hyperbranched polymers with the addition of vinyl phosphonic and other functional groups (e.g., aconitic acid, acrylic acid, maleic acid, and vinyl sulfonic) as scale inhibitors for preventing the formation of calcite and barite scales. Figure 9 shows the generalized structure of a hyperbranched polyethyleneimine with primary, secondary, and tertiary amine groups and the addition of vinyl monomers to primary and secondary amines. These authors found that all the hyperbranched inhibitors exhibited a satisfactory inhibition against both carbonate and sulfate scales. It should be noticed that among these hyperbranched polymers, phosphonate-derived polymers exhibited the best performance in inhibiting sulfate scale, whereas the aconate-derived polymer showed the best inhibition performance against carbonate scales. 

**Figure 10:**
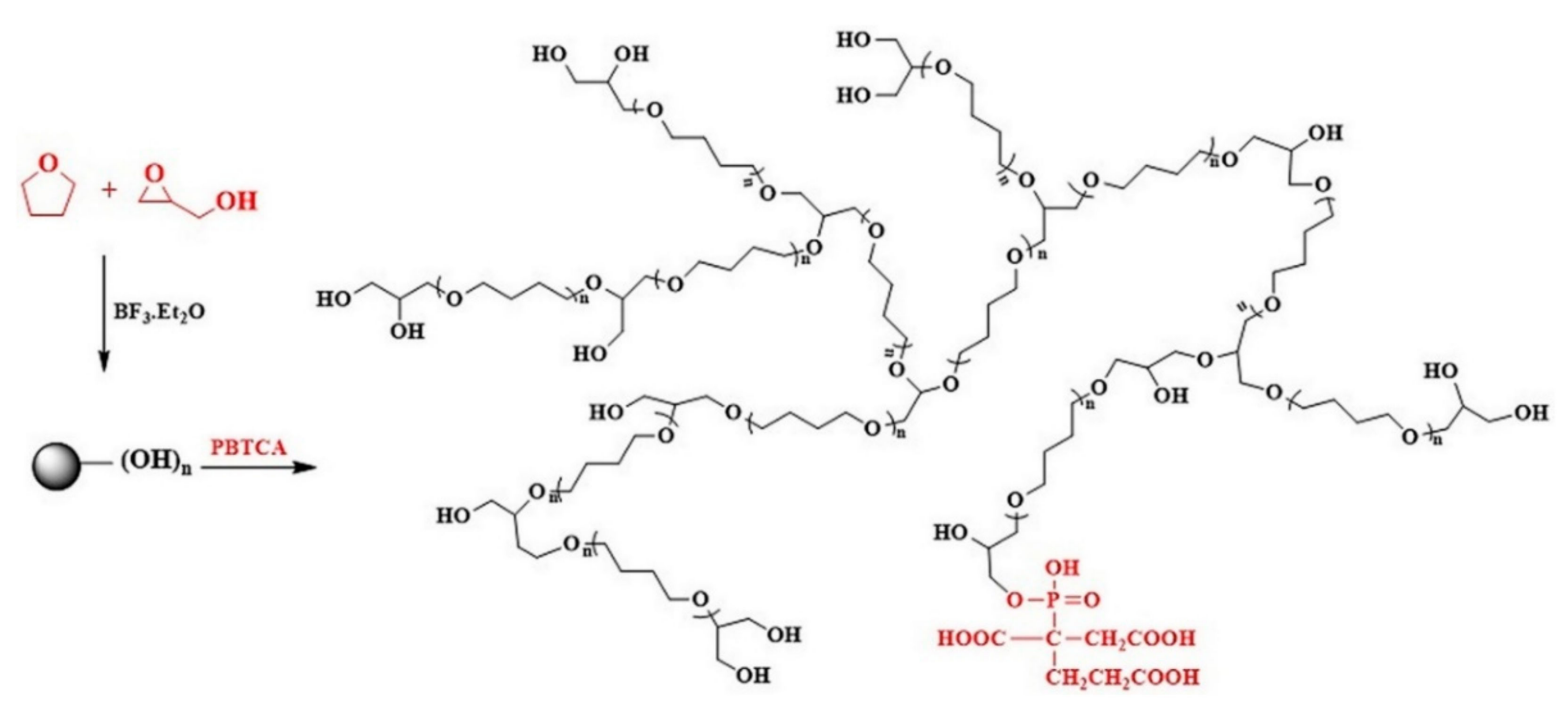
Synthesis approach of HBPs [123].

**Figure 11:**
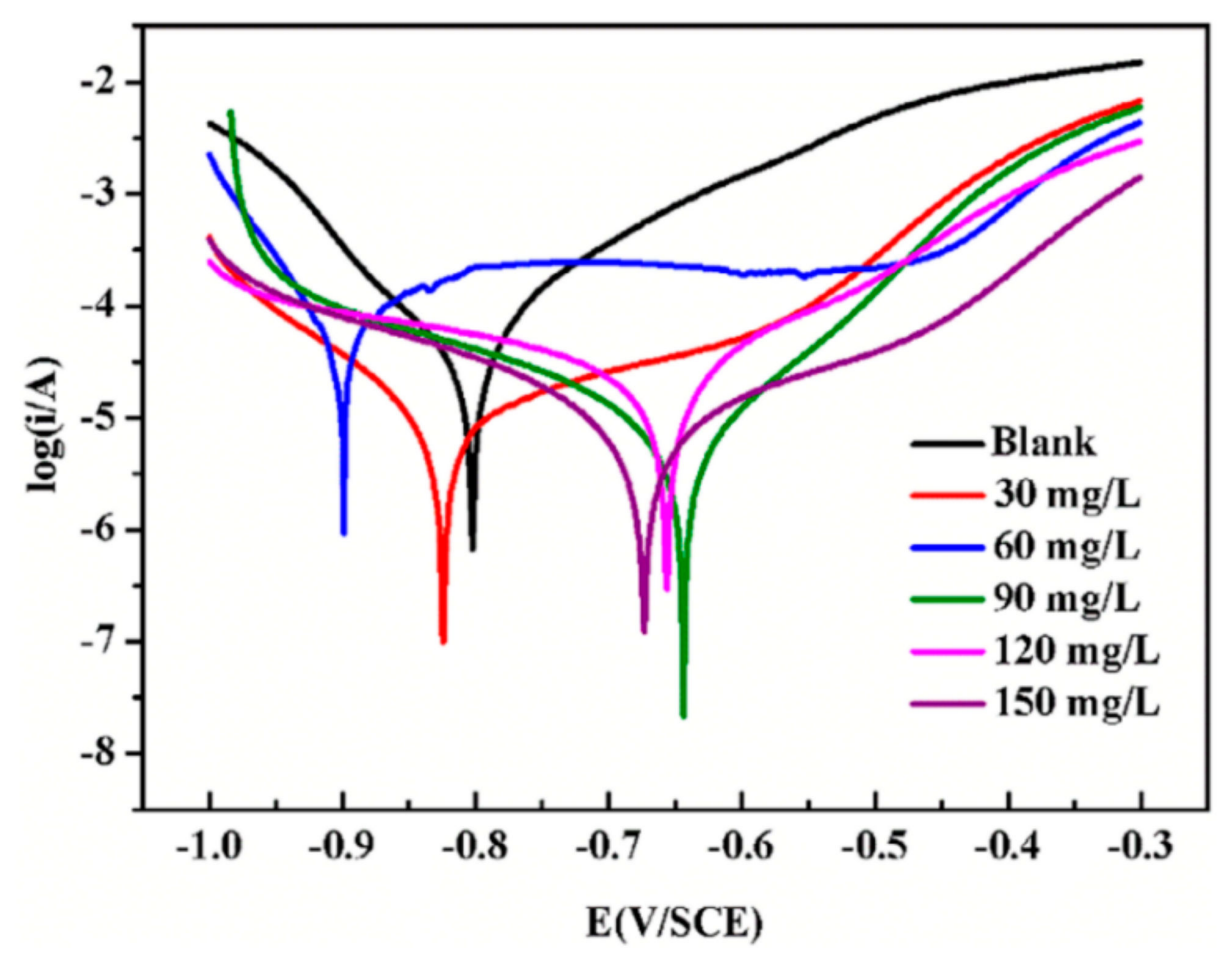
Potentiodynamic polarization curves recorded for the carbon steel electrode in 3 wt % NaCl solution containing different concentrations of HBP−3 [123].

In another study, Wang and his colleagues [123] reported the preparation of 2-phosphono-butane-1,2,4-tricarboxylic acid (PBTCA) modified hyperbranched polyether (HBP) as scale and corrosion inhibitors, as shown in Figure 10. The HBP chemical was prepared by cationic open-loop polymerization with tetrahydrofuran and glycidol. Carboxyl and phosphorus were then introduced to HBP by PBTCA to ameliorate the performance of corrosion and scale inhibition. PBTCA is a high-efficiency and thermally stable scale inhibitor due to its unique structure (-PO_3_H_2_ and -COOH), which is beneficial to enhance the adsorption of Ca^2+^ [124]. The results show that the scale inhibition efficiency of PBTCA-modified HBP (20 mg L^−1^) for CaCO_3_ and CaSO_4_ reached over 99% and 97% inhibition, respectively. This high inhibition efficiency was achieved by inhibiting the growth sites of calcium scale crystals and destroying the crystal structure of formed scale particles. At a chemical dosage of 150 mg L^−1^, PBTCA-modified HBP can result in a maximum corrosion inhibition efficiency of ca. 73%. As shown in Figure 11, polarization curves reveal that the fabricated HBPs acted as a mixed cathodic and anodic corrosion inhibitor. The adsorption experimental results suggest that the adsorption of HBPs on carbon steel surfaces follows a Langmuir-type adsorption isotherm. It is proposed that the functioning mechanism of corrosion inhibition by HBPs is the formation of a physical adsorption film on the surface of carbon steel. When the corrosion inhibitor was added, the corrosion potential of carbon steel moved to the positive pole and the corrosion current decreased, indicating that the corrosion process was inhibited. The inhibition efficiency of HBP-3 could reach more than 60%.

### 3.9. Other Terpolymer

**Figure 12:**
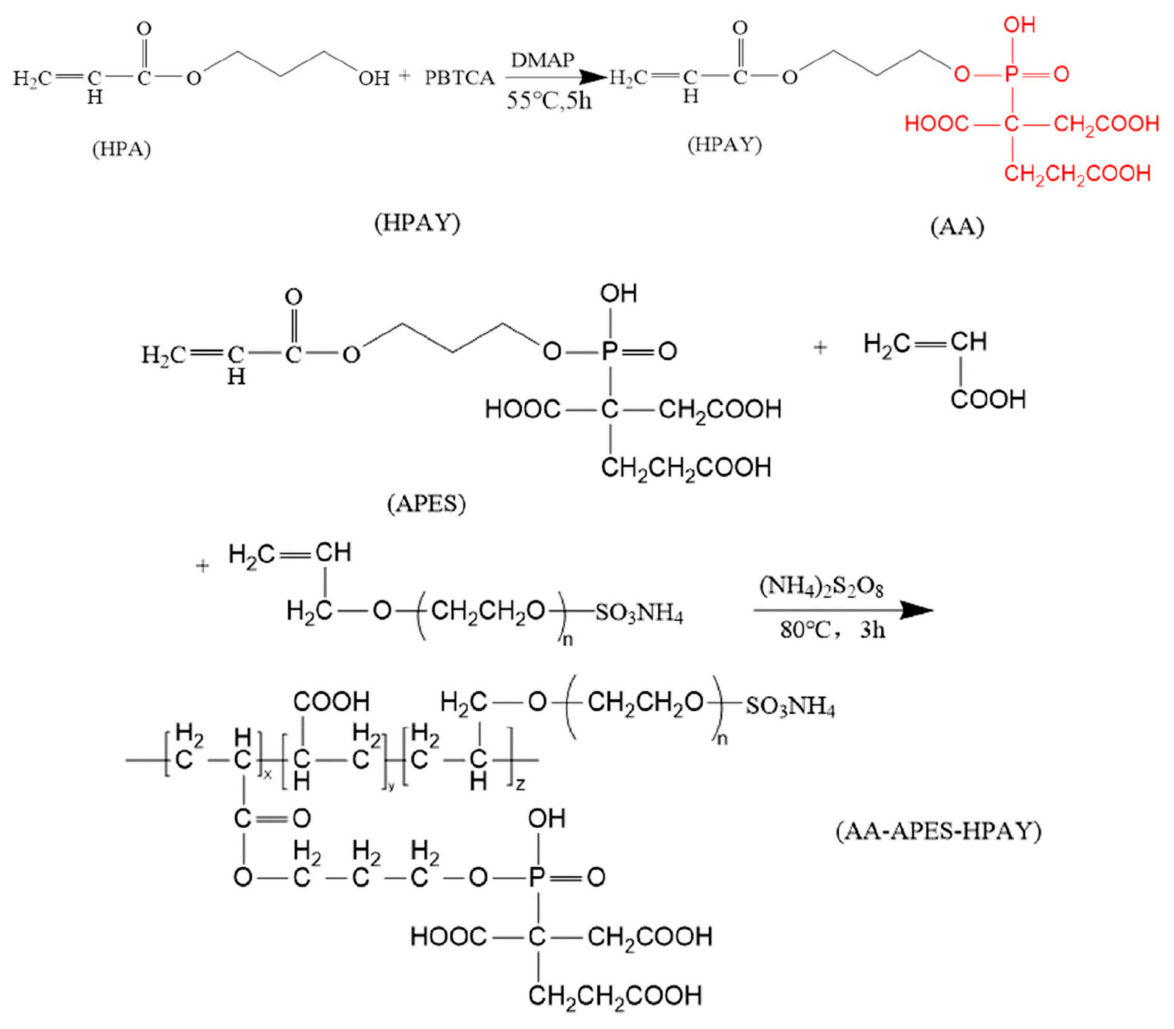
Synthesis of AA−APES−HPAY [128].

**Figure 13:**
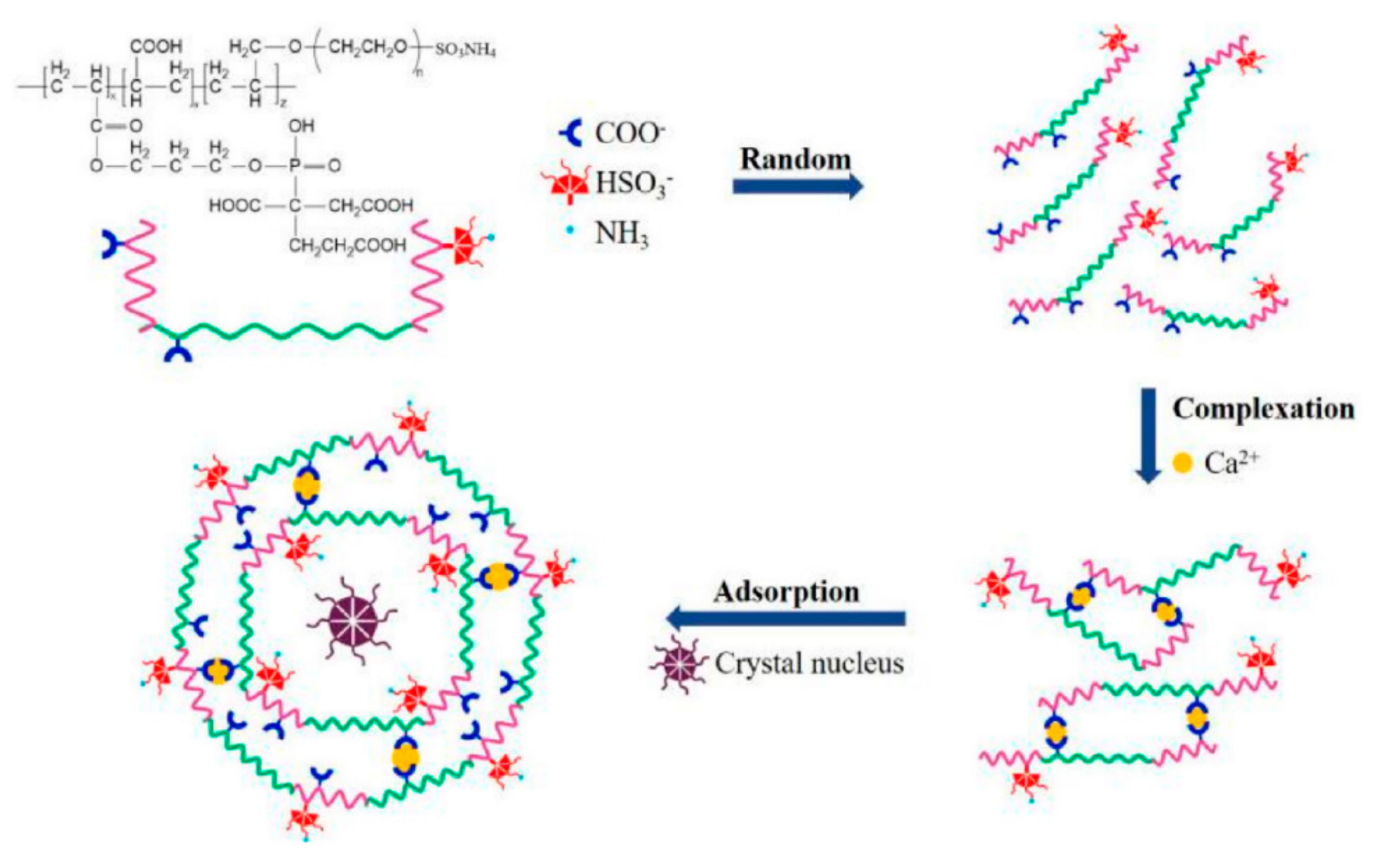
Schematic diagram of AA−APES−HPAY inhibition on calcium scales [128].

It is well known that synergistic co-additives with phosphorus embedded have been extensively investigated by many industries [125,126,127]. There are considerable benefits to combining phosphorus with other functional groups, such as carboxylic acid, sulfonic acid, and ether. Liu et al. [128] reported the synthesis of a new class of modified terpolymer (AA−APES−HPAY) as a scale inhibitor for oilfield applications. Figure 12 shows the synthesis route of this modified terpolymer. This hydrophilic terpolymer scale inhibitor was synthesized via free radical processes with acrylic acid (AA), hydroxypropyl acrylate modified by 2-phosphonobutane-1,2,4-tricarboxylic acid (HPAY), and allyl polyethoxyammonium sulfonate (APES) as monomers in aqueous solution, which contains a carboxylic acid group, phosphate group, sulfonic acid group, and ether bonds. According to these authors, experimental results show that when the AA−APES−HPAY level was at 21 mg L^−1^, more than 89% of the CaCO_3_ was inhibited, and the scale inhibition efficiency for CaSO_4_ could reach 92% with only 3 mg L^−1^ AA−APES−HPAY applied. In addition, these authors investigated the scale inhibition mechanism of the terpolymer and proposed the hypothesis that the modified terpolymer could control scale deposition via the mechanisms of adsorption, chelating dispersion, and electrostatic repulsion, as shown in Figure 13. However, based on the schematic diagram of AA−APES−HPAY inhibition of calcium scales, it shows that 10 molecules of inhibitor adsorb onto only one crystal nucleus, which is not compatible with the threshold (sub-stoichiometric) inhibition. A possible explanation could be that the modified terpolymer inhibits calcium scale formation by forming natural solid nanoimpurities rather than interaction with calcium salt nuclei during the prenucleation step [129]. Thus, additional studies are required to clarify the inhibition mechanism of this terpolymer inhibitor. 

The recently developed phosphorus-containing polymeric inhibitors include phosphorus end-capped polymers, grafted copolymers, hyperbranched polymers, etc. Table 4 summarizes the key properties, including efficiency and toxicity of these phosphorus-based polymers. 

## 4. Application for Oilfield Scale and Corrosion Control

The conventional method of adopting scale and corrosion inhibitors in an oilfield including continuous injection (through the annulus or spaghetti tubes), batch injection (e.g., tubing displacement), and squeeze treatment [61,130,131,132]. During oil and gas processing and production, pill solutions composed of combined scale inhibitor and corrosion inhibitor are usually injected together with other various chemical additives [133,134]. Among these methods, squeeze treatment is perhaps the most widely adopted approach in managing scale and corrosion threats at the downhole and reservoir [135,136]. During a squeeze treatment, the inhibitor solution or pill will be injected into the reservoir [137]. These inhibitors react with formation rock by adsorbing or precipitating onto rock material surfaces to fix the inhibitor in the formation pore space [138,139].

### 4.1. Conventional Squeeze Procedure and Ideal Squeeze Inhibitor Selection

**Figure 14:**
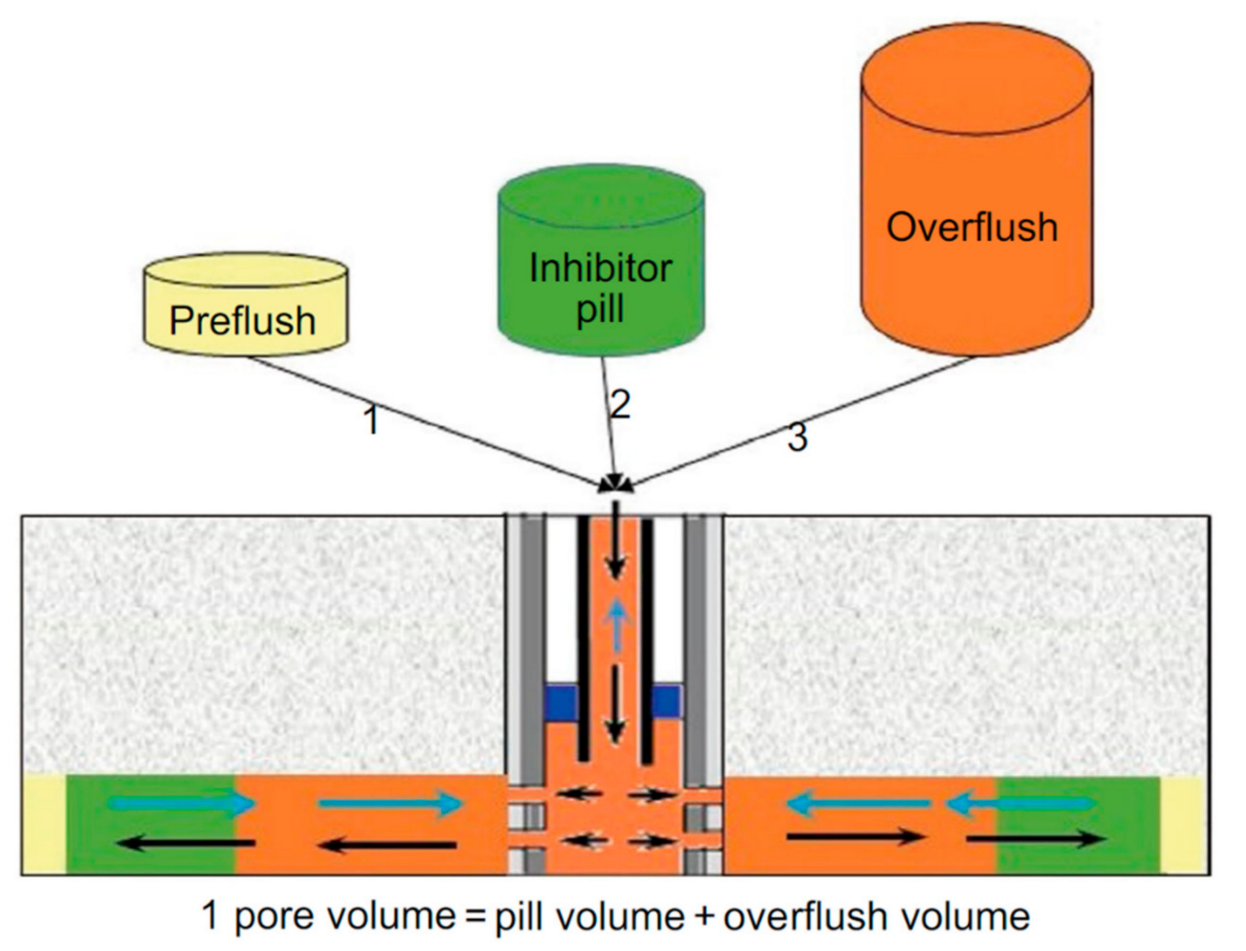
An illustration of a scale inhibitor squeeze treatment [137].

Typically, the injection sequence in a squeeze campaign usually follows (1) a preflush solution (acids, biocides, chelating agents, surfactants, etc.) to condition the reservoir before pill injection; (2) a volume of inhibitor pill solution (0.5–10% (*w*/*v*)) to be delivered through the production well into the wellbore; and (3) an overflush solution to push the injected inhibitor pill into deeper formation zone. The illustration of these three stages of conventional squeeze treatment is depicted in Figure 14. Following the squeeze treatment, scale inhibitor will be released and flowed back with the produced fluid to prevent scale along with the formation water. The determining factor for the success in a squeeze treatment is the longevity of squeeze lifetime, which is the time the inhibitor flows back at concentration required to prevent scale. Similar to the concept of squeeze treatment of mineral scale inhibitors, corrosion inhibitors can also be injected into the wellbore by a squeeze process in an attempt to create an alternative continual injection for corrosion control [89,140]. 

There have been intensive efforts to enhance the squeeze lifetime by adopting various inhibitor chemicals, particularly phosphorus-containing inhibitors [141,142,143]. For example, the phosphate ester group incorporated into polysulfonates shows good adsorption/desorption performances, which help retain inhibitors in the reservoir and prolong the treatment lifetimes [58]. The most widely used inhibitors for squeeze treatments includes phosphonates, polyacrylates, phosphinocarboxylics, and sulfonated polymers. The following are the main properties of an ideal squeeze inhibitor to be considered for squeeze treatments [18,135].

- Good inhibitory effectiveness at low levels of inhibitor concentrations, typically on the order of 1–50 mg L^−1^;
- Good compatibility with seawater, formation water, and other chemical additives for the application in oilfield flow assurance;
- Good adsorption/desorption properties allowing the long-term slow release of chemicals into production water at concentrations above the required scaling prevention level;
- High resistance to temperatures and pressures encountered downhole. It is not desirable to undergo thermal degradation under downhole conditions;
- More environmentally friendly than phosphonates;
- Balance between cost-effectiveness and affordability.

### 4.2. Retention/Release Mechanism of Inhibitor in the Formation

During the squeeze treatments, the chemical inhibitor can be retained in the reservoir formation near producing wells by either adsorption or precipitation mechanisms (or both). “Adsorption” squeezes often occur in certain noncarbonate (sand) reservoirs by injecting a neutralized form of phosphonate pill, whereas the “precipitation” squeeze is generally performed by injecting an acidic phosphonate pill into a carbonate-rich formation to dissolve carbonate-rich formation rock and form the precipitation of calcium phosphonate [144]. The mechanism of inhibitor retention/release in the formation is determined by reactions of the inhibitor with formation rock. As noted above, PPCA is one of the most commonly used polymeric inhibitors in oilfields and is often applied in a “precipitation” squeeze treatment [145]. The sparingly soluble Ca–PPCA complex will be formed during the reaction processes between PPCA and dissolved calcium ions from the rock. The solubility of this complex and the kinetics of the dissolution are the two main factors that determine the retention and release of PPCA in squeeze treatment processes [146,147]. The PPCA inhibitor complex with Ca^2+^ is described as acrylic acid monomer (A), which can make extensions for different lengths of polymer chain with the following reaction scheme [68]:

$$\mathrm{HA}↔H^{+}+A^{-}\;\;\;\;\;\;\;\;\;\;\;\;\;K_{H}=\frac{\left\{H^{+}\right\}\left(A^{-}\right)}{\left(HA\right)}$$

$$\mathrm{Ca}\;(A\;\cdot \cdot \cdot A)\;↔\;{\mathrm{Ca}}^{2+}+{\left(A\;\cdot \cdot \cdot A\right)}^{2-}\;\;\;\;\;K_{\mathrm{Ca}}=\frac{\left(Ca^{2+}\right)\left(A\cdots A^{2-}\right)}{\left[Ca\left(A\cdots A\right)\right]}$$

Xiao et al. [68] reported a set of substituent constants of PPCA under realistic downhole conditions, which could be used to analyze the equilibria of PPCA in liquid solutions, at given temperature, pH, ionic strength, and total Ca^2+^ concentration conditions. These authors also concluded that Ca–PPCA demonstrated a lower solubility than that of Ca–BHPMP (bishexamethylenediamine penta (methylene phosphonic acid)) and higher than that of Ca–NTMP (nitrilo tris (methylenephosphonic acid)) [146]. The molecular weight plays a critical role in the solubility of Ca–PPCA precipitates [148]. For example, Andrei and Gagliardi [149] found that the molecular weight of redissolved fractions of soft Ca–PPCA precipitate was much higher than hard-solid complex precipitate.

### 4.3. New Phosphorus-Based Inhibitors Used in Squeeze Treatment

**Figure 15:**
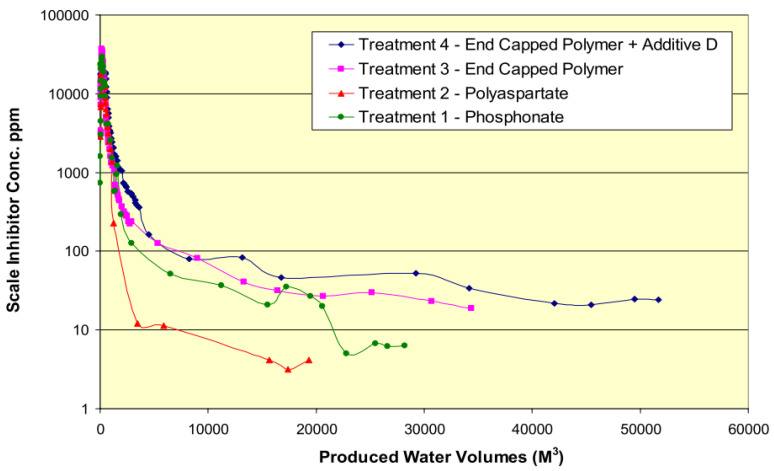
Comparison of inhibitor return profiles at different field treatments [152].

Many polymeric inhibitors employed for squeeze treatment have some inherent disadvantages, such as lower adsorption/desorption performances compared with phosphonates [150,151]. However, with the introduction of novel phosphorus-based polymers, the gap in terms of squeeze treatment performance is rapidly narrowing [41,100]. As noted above, many new phosphorus-based chemical inhibitors have been synthesized and evaluated in the laboratory to fulfil the requirements of an ideal squeeze inhibitor. However, there are limited reports on the application of these chemicals for oilfield squeeze treatment. Selle et al. [152] reported a phosphorus end capped polymer deployed to control scale in the Haltenbanken area offshore mid-Norway. As shown in Figure 15, the phosphorus end capped polymer was performed in Treatment #3, which was able to retain above 20 mg L^−1^ within approximately 35,000 m^3^ of the production brine. This indicated that the volume of brine protected is much higher than those for phosphonate (Treatment #1) and polyaspartate (Treatment #2) inhibitors. In addition, with a multi-functional additive in preflush, adsorption property of the phosphorus end capped polymer could be significantly enhanced. The effective protection could reach approximately 52,000 m^3^ of the production brine, indicating a promising potential for a prolonged squeeze lifetime with this new phosphorus end capped polymer.

**Figure 16:**
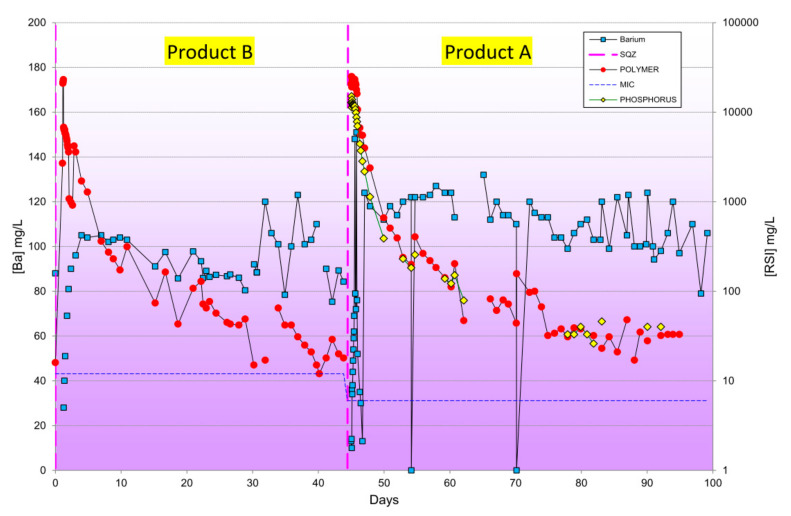
Field returns of incumbent inhibitor Product B and the subsequent field trial with Product A on Well E-22 [41].

In another study, following successful development of phosphorus tagged polymeric inhibitors [100], Todd et al. [41] adopted these phosphorus-functionalized polymeric inhibitors in sequential field trials in a North Sea carbonate reservoir under harsh BaSO_4_ scaling and highly naturally fractured conditions (Figure 16). The trial results showed that the induction period in the squeeze treatment was longer for Product A (a novel phosphorus functionalized sulphonated copolymer) than for Product B (a standard sulphonated copolymer), and it appears that the scaling amount is lessened in the later stages of the squeeze lifetime of Product A. These results indicate that the phosphorus functionalized inhibitor could provide improved scaling control performance compared to the incumbent product, especially under harsh operating conditions.

**Figure 17:**
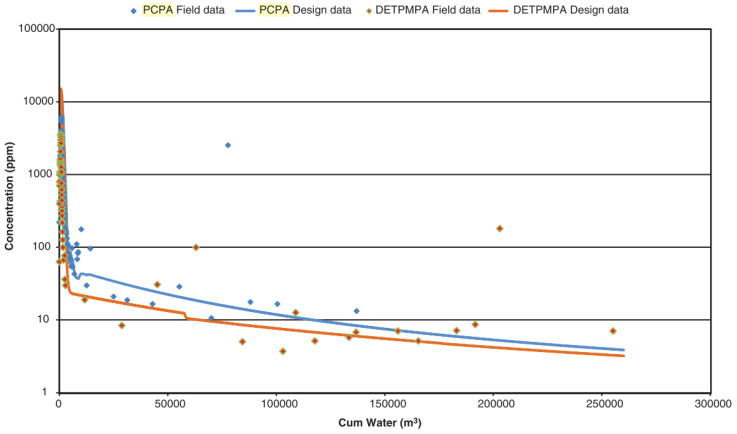
Field returns of incumbent inhibitor Product B and the subsequent field trial with Product A on Well E-22 [153].

Jordan and his colleagues [153] reported an environmentally acceptable phosphorus-containing polymer amine (PCPA) chemical for squeeze treatments in the North Sea region. The adsorption properties of the modified polymer can be improved by incorporating amine groups into the anionic polymer, which is comparable to that of phosphonate (Figure 17). It shows that PCPA can provide a much longer return period relative to conventional inhibitor chemicals such as DTPMP. The most commonly adopted DTPMP inhibitor was replaced by PCPA in their work, leading to a significant reduction in the environmental impact of these operations.

With respect to the application for oilfield scale and corrosion control, phosphorus-based inhibitors demonstrate their advantages in terms of efficacy, prolonging the treatment lifetime, and being environmentally friendly in squeeze treatment. 

## 5. Conclusions and Future Perspectives 

Mineral scale deposition and metal corrosion are among the major concerns related to operational safety and production efficiency during oil and gas production. Deployment of chemical inhibitors is an effective and economical means to reduce and control these threats. Although conventional phosphonate-based inhibitors generally exhibit improved inhibition and adsorption/precipitation properties compared with other polymeric inhibitors, increasing environmental regulations and discharge limitations on phosphonates make them more and more undesirable to apply in the field. In the past few decades, various new classes of low-toxic/nontoxic and biodegradable phosphorus-containing polymers have been reported to improve the efficiency functionalities of these chemicals. This review article is mainly focused on the scale and corrosion inhibition mechanism, synthesis and laboratory evaluation, as well as application in oilfield squeeze treatment of various phosphorus-containing polymeric inhibitors in the oil and gas industry. In spite of the significant development so far, there is a need to design novel green polymeric inhibitors with a good inhibition efficiency, good thermolability, non-toxicity, and cost-effective properties to improve the applicability of these chemicals for field applications. In addition, the relationship between inhibition mechanism and polymer structure as well as inhibition performance should be systematically evaluated, which will provide theoretical guidance for the development of new classes of chemical inhibitors. Finally, the speciation and solubility of new phosphorus-based polymer inhibitors should be fully investigated to provide fundamental understanding on the release of the inhibitors after field chemical squeeze treatment.

## Figures and Tables

**Table 1:** Summary of the common scale inhibitors.

Type	Scale Inhibitor	Advantage	Disadvantage
Polyphosphates	Sodium tripolyphosphate, Sodium hexametaphosphate	Effective scale and corrosion inhibition	Low solubility; lower thermal stabilities than phosphonates; Easily hydrolysed into orthophosphates to form insoluble calcium salts
Phosphonates	BHPMP, DTPMP, HEDP, NTMP, and PBTC	Good scale and corrosion inhibition; Good adsorption	Poor biodegradability; Less thermally stable than polymeric species; Poor compatibilities with the production system
Phosphino-polycarboxylic acid	PPCA	Excellent calcium carbonate and calcium sulfate inhibition; Great barium sulfate inhibitor; High calcium tolerance and halogen resistant; Hydrothermally stable	Less adsorption than phosphonates
Polyacrylates	PAA	Good scale inhibition; Good dispersion	Poor biodegradation; Poor adsorption
Polymaleates	PMA	Fairly biodegradable	Poor adsorption
Polyepoxysuccinic acid	PESA	Highly biodegradable; Good corrosion inhibition	
Polyaspartates	PASP	Highly biodegradable	

**Table 2:** Summary of common phosphorus-based polymers.

Phosphorus-Based Polymers	Abbreviation	Mol. Mass (Dalton)	Structure
Polyamino polyether methylene phosphonic acid	PAPEMP	600	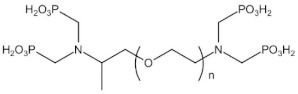
Phosphono carboxylic acid	POCA	2000	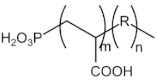
Phosphino-polycarboxylic acid	PPCA	3800	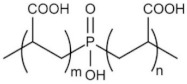
Maleic acid–sodium q-styrenesulfonate copolymer	MAc-SS	1.86 × 10^5^	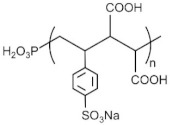
*N*-phosphonomethylated amino-2-hydroxypropylene polymer	PMPA	300–5000	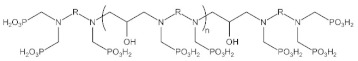

**Table 3:** P-tagged copolymer.

Structure	Notes
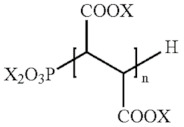	wherein the scale inhibitor comprises a phosphonic acid terminated polymer according to formula [104].
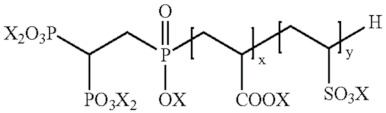	wherein X corresponds to H or an anion, and x + y is an integer between 2 and 500 [104].
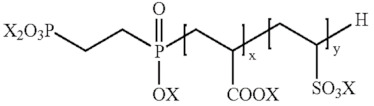	wherein X denotes H or an anion, and x + y is an integer between 2 and 500. [104]
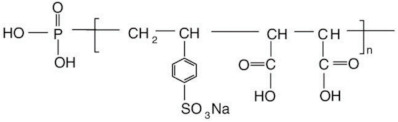	[97]
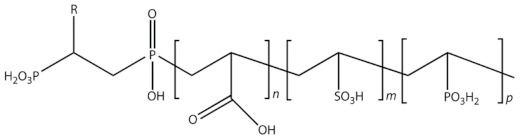	wherein R represents H or PO_3_H_2_, and m, n, and p can be either zeroor any number [58].

**Table 4:** Properties of recently developed phosphorus-based polymers.

Phosphorus-Based Polymer Inhibitor	Efficiency	Toxicity ^a^	Other Properties
Phosphino poly carboxylic acid (PPCA) [95]	Complete gypsum inhibition at SI = 0.31 at any pH and T; 24% inhibition with NaCl at an extremely high SI of 1.47	N.A. ^b^	N.A. ^b^
P-tagged copolymer [97]	98.2% inhibition effect on CaCO_3_ with 16 ppm inhibitor at 80 °C for 10 h	Environmentally safe	N.A. ^b^
P-tagged copolymer [100]	Greater inhibition performance than DTPMP and sulphonated copolymer	N.A. ^b^	Excellent thermal stability; Comparable Ca tolerance relative to standard sulphonated copolymer
P-tagged copolymer [102]	Similar inhibition performance with the best conventional polymeric scale inhibitor under extreme conditions	N.A. ^b^	Enhancement of adsorption property; Stable at least 200 °C; 30% biodegradation in 28 days in seawater by OECD306 test
Phosphonated polyetheramines [108]	Improved inhibition performance against both calcite and barite compared to common commercial phosphonated inhibitors	Environmentally friendly	Superior calcium tolerance; good thermal stability at 130 °C 47% biodegradation in 28 days in seawater by OECD306 test
Phosphonated aliphatic polycarbonates [109]	Better inhibition performance against calcite and barite compared to carboxylated homopolymer	Environmentally friendly	Enhancement of water thermal stability; 36% biodegradation in 28 days in seawater by OECD306 test
Phosphonated polyaspartic acid [44]	Excellent calcite scale inhibition property under high pressure high temperature conditions	Environmentally friendly	Desirable thermal stability under harsh oilfield conditions (at 130 °C for 7 days) compared to PASP and other modified compounds; Calcium tolerance ability with Ca^2+^ ions up to 100 mg L^−1^
Grafted copolymer [117,118]	Able to control multiple scales in any process field, in particular under harsh conditions; Poor inhibition performance with brines in high scaling tendency systems	N.A. ^b^	N.A. ^b^
Dendrimeric or hyperbranched polymers [123]	99% CaCO_3_ and 97% CaSO_4_ inhibition, respectively at 20 mg L^−1^; ca. 73% corrosion inhibition efficiency at 150 mg L^−1^	Environmentally friendly	N.A. ^b^
Other terpolymer [128]	89.2% CaCO_3_ inhibition at 21 mg L^−1^; 92.4% CaSO_4_ inhibition at the 3 mg L^−1^	N.A. ^b^	Good hydrolytic stability

^a^ The toxicity of inhibitor was described in the corresponding references. ^b^ The toxicity or other properties of inhibitor was not noted in the corresponding references.

## Nomenclature

AA	acrylic acid
APES	allyl polyethoxyammonium sulfonate
BHPMP	bishexamethylenediamine penta (methylene phosphonic acid)
DTPMP	diethylenetriamine penta (methylene phosphonic acid)
EOR	enhanced oil recovery
HBP	hyperbranched polyether
HEDP	1-hydroxyethane-1,1-bis (phosphonic acid)
HPAY	hydroxypropyl acrylate modified by 2-phosphonobutane-1,2,4-tricarboxylic acid
MAc-SS	maleic acid–sodium q-styrenesulfonate copolymer
NTMP	nitrilo tris (methylenephosphonic acid)
PAA	polyacrylic acid
PAPEMP	polyamino polyether methylene phosphonic acid
PASP	polyaspartic acid
PBTC/PBTCA	2-phosphono-butane-1,2,4-tricarboxylic acid
PCA	polycarbonates
PCPA	phosphorus-containing polymer amine
PEG	polyethylene glycol
PESA	polyepoxysuccinic acid
PHOS	phosphonic acid
PMA	polymaleic acid
PMPA	phosphono methylated polyamine
POCA	phosphono carboxylic acid
PPCA	phosphino-polycarboxylate
SEM	scanning electron microscopy
SPCA	sulfonated polycarboxylic acid
VDPA	vinylidene diphosphonic acid
VPA	vinyl phosphonic acid
